# Proton First: Rationalizing a Proton Transfer in a Protein‐Fragment Complex

**DOI:** 10.1002/cmdc.202500244

**Published:** 2025-09-14

**Authors:** Helge Vatheuer, Jonas Paulus, Lisa Johannknecht, Gerald Keller, Rebecca Maria Ziora, Lukas S. Stelzl, Paul Czodrowski

**Affiliations:** ^1^ Department of Chemistry Johannes Gutenberg University Duesbergweg 10–14 55128 Mainz Germany; ^2^ Institute of Molecular Physiology Johannes Gutenberg University Hanns‐Dieter‐Hüsch‐Weg 17 55128 Mainz Germany; ^3^ Institute for Quantitative and Computational Biosciences (IQCB) Johannes Gutenberg University Mainz 55128 Mainz Germany; ^4^ Institute of Physics Johannes Gutenberg University Staudingerweg 7 55128 Mainz Germany

**Keywords:** isothermal titration calorimetry, molecular dynamics simulations, protein crystallography, protein kinase A, proton transfers

## Abstract

A combination of experimental and theoretical approaches is used to decipher the molecular recognition event of benzoic acid complexed with protein kinase A. The publicly known crystal structure suggests the protonated form of benzoic acid to be complexed with Protein Kinase A. Such a protonation pattern of is unlikely for benzoic acid in aqueous environment and must be induced by complexation to protein kinase A. Unfortunately, isothermal titration calorimetry does not reveal any binding event, which may be due to low affinity. However, Poisson–Boltzmann calculations and molecular dynamics simulations strengthen the initial hypothesis of a protonated benzoic acid binding to protein kinase A.

## Graphical abstract

We combine experimental and theoretical approaches to investigate benzoic acid binding to Protein Kinase A. The crystal structure shows benzoic acid protonated in the complex. Isothermal titration calorimetry (ITC) fails to detect binding, likely due to low affinity. Molecular dynamic (MD) simulations indicate that the protonation state has indeed a huge impact on the binding behavior, matching the previous results.

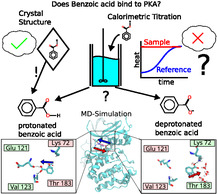

## 1 Introduction

Fragments are small molecules for which the ”Rule Of Three” (RO3), that is, a molecular weight of ≤300 Da, a number of hydrogen bond donors and acceptors of ≤3, and a cLogP of ≤3, is frequently applied during their selection.^[^ ^1^ ^]^ Fragment‐based approaches, that is, screening campaigns based on fragments fulfilling the RO3 criteria, have recently emanated into approved drugs such as vemurafenib and venetoclax.^[^ ^2^ ^]^

As part of the fragment‐based drug design process, the molecular recognition event is carefully inspected (usually by human experience) to determine the crucial protein‐ligand interactions. Atomistic molecular dynamics (MD) simulations can give important insights into the interactions drug target such as kinases with ligands.^[^ 3, 4, 5 ^]^ As part of this inspection, possible protonation changes upon complexation can be revealed. For several drugs and advanced drug candidates, such a protonation event is known for several protein families and different ligand chemotypes.^[^ 6, 7, 8 ^]^ These effects are usually not considered even though they apply equally well along the fragment‐based drug design process which might lead, for example to an incorrect setup of a pharmacophore for a virtual screening campaign resulting in no hits or misleading docking hits which assume a false protonation pattern.

**Figure 1:**
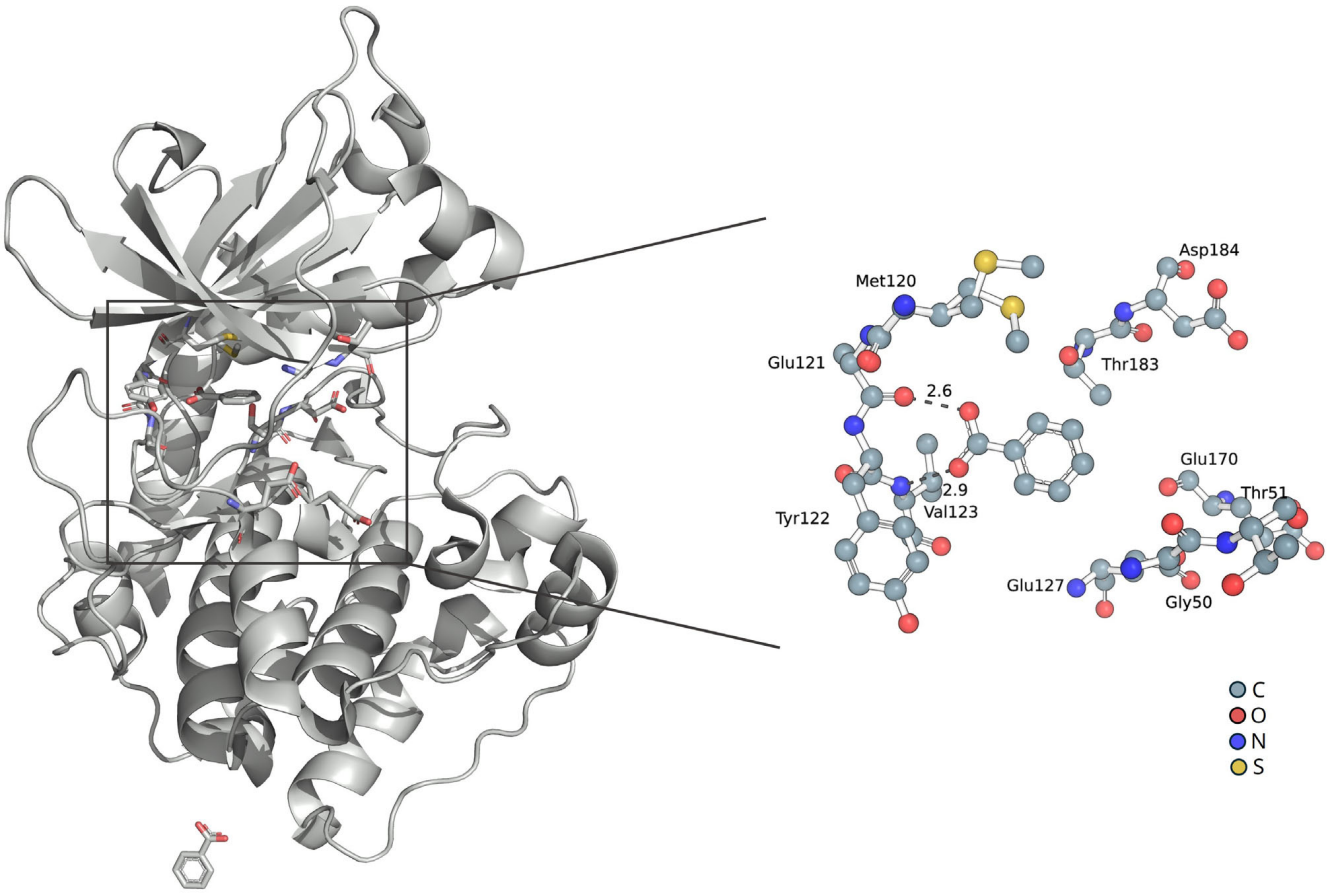
PKA‐benzoic acid complex (PDB entry: 6SNN) with detailed view of the hinge binding site; distances in Å.

In a crystallographic fragment screen in the ATP‐site of protein kinase A (PKA), benzoic acid was identified as a binder to its hinge region.^[^ ^9^ ^]^ The interaction pattern (see **Figure** **1**) of benzoic acid complexing PKA suggests that the protonated form of benzoic acid binds to PKA. This would only occur if the pKa value of the carboxylic acid group of benzoic acid, which ranges from 4.0 to 4.2 in its unbound state in aqueous solution (as noted in https://tools.czodrowskilab.org/), shows a significant shift in pKa value upon binding to the protein PKA, resulting in a protonation event during the complexation.

Such a protonation event can be detected by isothermal titration calorimetry (ITC), for which two different approaches exist for low‐affinity binders such as fragments: low *c* value titration and displacement titration (see Experimental Section).

We were curious to test the hypothesis that benzoic acid binds in a protonated (unusual) fashion to the ATP site of PKA and if ITC could reveal this perturbed protonation pattern. We also ran implicit solvent pKa calculations to study the pKa values of the benzoic acid in the ATP pocket. Lastly, we ran atomistic MD simulations to study the stability of the neutral versus charged benzoic acid complexed with PKA. As part of this study, we also investigated the key interaction motifs for benzamide complexed with PKA for which a crystal structure is available as well.^[^ ^9^ ^]^

## 2 Results

Fasudil was used as high‐affinity PKA inhibitor and displacement ligand. As a control for both the displacement titration and to verify the activity of the protein PKA, PKA was titrated with fasudil alone. Although no thermodynamic parameters have been published for human PKA, they have been published for the homologous variant from *Cricetulus griseus*, which differs only at six terminal amino acids.^[^ ^10^ ^]^

**Figure 2:**
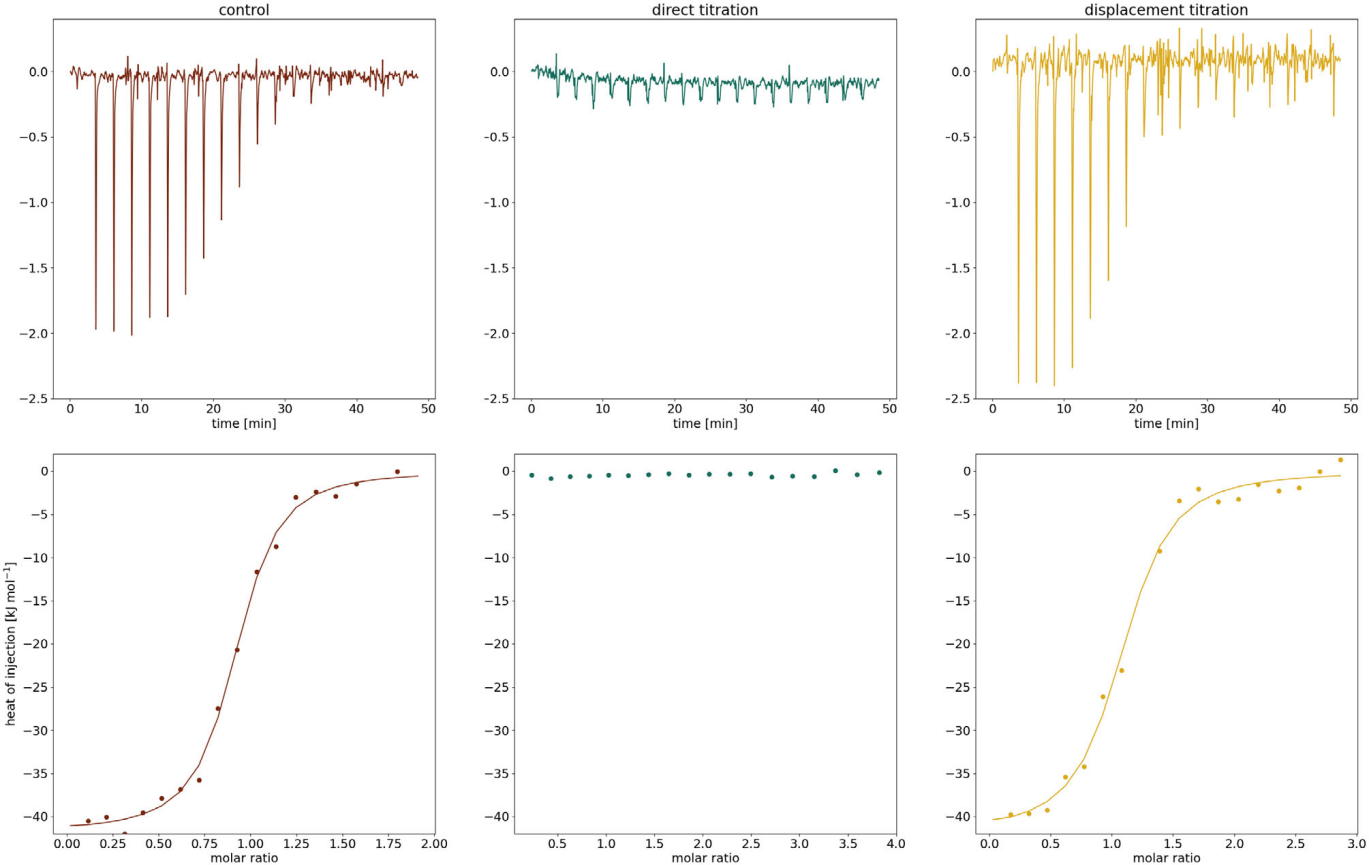
Overview of the different ITC experiments to investigate the PKA–benzoic acid interaction. Upper row: raw thermograms. Bottom row: integrated data of the heat signals observed for the measurements and corresponding fitted lines.

The result of this control measurement revealed a stoichiometry of *N* = 0.9, meaning that most of the protein is intact and active (**Figure** **2**, left column). The overall ΔG of −37.3 kJ mol^−1^ is also in close proximity of the hamster variant (ΔG = −36.1 kJ mol^−1^).^[^ ^10^ ^]^

In order to test the benzoic acid fragment, we firstly performed a direct titration of PKA and benzoic acid (Figure 2, middle column). The used concentrations were 140 μM for the protein and 2.8 mM for the benzoic acid, respectively. Unfortunately, a binding signal could not be detected.

Next, a displacement titration (Figure 2, right column) was carried out. For this purpose, the protein (c = 20 μM) was incubated with the fragment (c = 4.2 mM) prior to the titration with strong binding fasudil (c = 300 μM), the displacing ligand. The differences in the thermodynamic profiles of PKA and fasudil and in the displacement titration are so small that they are within the error of the experiments. Nonetheless, one should keep in mind that a difference in the free binding energy can only be measured if the binding enthalpies siginificantly differ from each other.

We then ran implicit solvent Poisson–Boltzmann calculation for the PKA–benzoic acid complex. Given such a calculation, the pKa values of the titratable groups of PKA and the benzoic acid are estimated. The ΔpKa of the benzoic acid carboxyl group was 1.2, thus shifting from 4.2 (experimental value) to 5.4 (protein‐bound). No titratable group of PKA shows a significant pKa shift (more than one log unit) which could give rise to a proton transfer (i.e. a protein residue which becomes protonated or deprotonated upon complexation with benzoic acid). This, together with the pH shift due to the composition of the solution, makes a protonated benzoic acid molecule very likely.

We then inspected the composition of the soaking solution which was used for the crystal structure determination: the details are given in **Table** **1**. This solution is primarily composed of organic solvents —methanol, DMSO and MPD— which together constitute more than 50% (v/v). We hypothesized that due to organic solvents, the pH value of the entire solvent system is lowered (compared to physiological conditions). Therefore, this soaking solution was reconstituted and its pH was measured. As expected, the pH value decreases 1.6 units, from 6.9 to 5.3. We also measured the pKa value of benzoic acid in this buffer and obtained a value of 4.1 which results in almost 10% occurrence of the protonated form benzoic acid.

**Table 1:** Composition of the solution used for soaking benzoic acid into the PKA crystal.

Components	v/v [%]
100 mM MES‐BIS‐TRIS pH 6.9	–
75 mM LiCl, 0.03 mM Mega8, 1 mM DTT	60
0.1 mM EDTA, 23% (v/v) MeOH	–
MPD	30
1 M benzoic acid in DMSO	10

**Figure 3:**
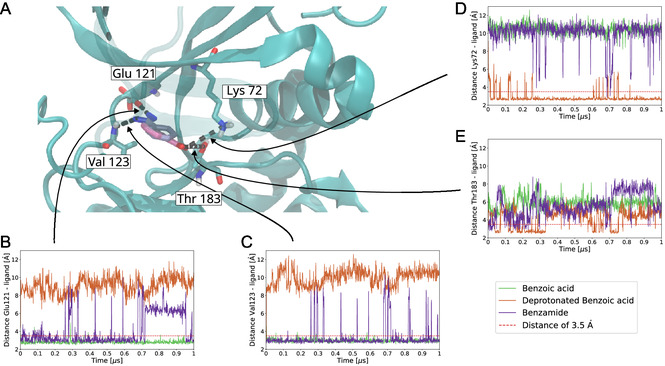
Hydrogen bonds between ligands and the PKA binding site in atomistic MD simulations. A) Close‐up look to the binding site of PKA, the four amino acids that form hydrogen bonds with PKA are highlighted. The distance of the amino acids to the ligand's hydrogen bond donor/acceptor over time are shown for B) Glu121, C) Lys72, D) Val123, and E) Thr183. The benzoic acid is shown in dark and light blue while the deprotonated benzoic acid is shown in red and pink. The dark blue and red part of the ligand are the functional carbonate group and the light blue and pink parts are the benzene ring. Potential hydrogen bond are indicated by black dashed lines. The protein structure from the simulation of PKA bound to protonated benzoic acid is shown. Some parts of the protein are shown transparently to enhance visual clarity. Solvent atoms are omitted for clarity.

This motivated us to pursue MD simulations to more comprehensively elucidate how the benzoic acid fragment binds to PKA. We started MD simulations with the binding pose from X‐ray crystallography (PDB entry: 6SNN) and simulated the benzoic acid in its protonated and deprotonated state. The overall structure of PKA with protonated benzoic acid and unprotonated benzoic acid does not change and remains close to the X‐ray structure as judged by computing the root mean square distance to the X‐ray structure (Figure S1C,D, Supporting Information). However, protonation of the benzoic acid fragments affects the binding pose of the fragment. The protonated form of the benzoic acid fragment is stable in its X‐ray determined binding pose (**Figure** **3**A). Over the course of a 1 μs simulation protonated benzoic acid maintains two hydrogen bonds (Figure S4A, Supporting Information), namely with Glu 121 and Val 123. The backbone carbonyl of Glu 121 interacts with the hydrogen of COOH group (Figure 3B). This interaction is stable throughout the simulation. The distance between oxygen and hydrogen atoms remains below the threshold of 3.5 Å, which is used to define hydrogen bonds. Similarly, the amide proton of Val 123 interacts with the carbonyl oxygen of the protonated benzoic acid and the oxygen hydrogen distance is below the threshold throughout the simulation (Figure 3C). The importance of the interactions of Glu 121, Lys 72, and Thr 183 with the protonated benzoic acid fragment are also highlighted by molecular mechanics / Poisson–Boltzmann surface area (MM/PBSA) calculations^[^ 11, 12, 13 ^]^ (Figure S4, Supporting Information).

**Figure 4:**
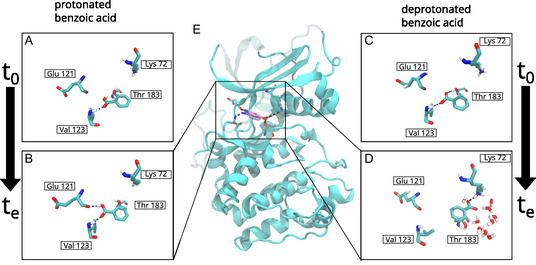
Atomistic MD simulation resolve conformational dynamics and preferred conformations of benzoic acid and deprotonated benzoic acid at physiological conditions. A) The initial state from the benzoic acid. B) Representative snapshot after equilibration ($t_{e}$). C) The initial state of the deprotonated benzoic. D) The binding pose of the ligand orientation after equilibration. In the middle, E) the entire simulated protein is shown without water and ions. The benzoic acid is shown in dark and light blue while the deprotonated benzoic acid is shown in red and pink. The dark blue and red part of the ligand are the functional carboxylate group and the light blue and pink parts are the benzene ring. Some parts of the protein are shown in a transparent representation for visual clarity.

By contrast, the deprotonated benzoic acid fragment rotates away from its original binding pose (Figure 1) and stays in this new orientation as indicated in Figure 3A, where the functional group of the deprotonated fragment can be seen to be rotated by 180° from the binding pose of protonated benzoic acid. Most of the time deprotonated benzoic acid maintains only a single hydrogen bond with PKA (Figure S4B, Supporting Information). Due to the reorientation of the carboxylate group, the carboxylate group is too far away from Glu 121 and Val 123. No hydrogen bonds are formed with Glu 121 and Val 123 (Figure 3B,C). However, in its new binding pose the deprotonated benzoic acid fragments interacts with the side chain of Lys 72 (Figure 3D). A hydrogen bond of the carboxylate group of the deprotonated fragment and the side chain hydroxyl group of Thr 183 breaks and reforms but is stable for extended trajectory segments, with bond stable for durations of more than 100 ns at around 200 ns of simulation, but breaks at around 800 ns of simulation time (Figure 3E). Importantly, the carboxylic group appears to drive the rearrangement and starts to interact with water molecules rather than the PKA (**Figure** **4**D). MM/PBSA calculation also show that the interactions of PKA residues with deprotonated benzoic acid are not favorable in general (Figure S6, Supporting Information).

Since MD simulations afford us the opportunity to straightforwardly extend our investigation to additional compounds and as a control for our simulations, we also simulated the complex of PKA with benzamide. The benzamide fragment has been shown by X‐ray crystallography to form similar interactions, in particular hydrogen bonds to PKA. Consequently, we expect this fragment to behave similar to the protonated benzoic acid fragment.^[^ ^9^ ^]^ Two hydrogen bonds between the protein and the fragment that are binding the protonated benzoic acid are largely maintained throughout the 1 μs trajectory (Figure S4C, Supporting Information). The conformation of PKA remained close to the X‐ray structure of PKA bound to benzamide in the simulation. MD simulations reveal also subtle differences between the two ligands (Figure 3B‐E). The carbonyl group of Glu 121 is a hydrogen bond acceptor and in this way interacts with the functional group of the ligand (Figure 3B). However, the hydrogen bond is less stable than for protonated benzoic acid and is broken after 0.7 μs of simulations before it forms again after 0.9 μs of simulations. The amide proton of Val 123 engages in a hydrogen bond with the carbonyl group of benzamide. This hydrogen bond is broken only for very short periods and reforms immediately, but is overall less stable than the hydrogen bond between protonated benzoic acid and Val 123. Like in the simulations with protonated benzoic acid, no stable hydgrogen bond is formed to Lys 72 and Thr 183, although the side chain of Thr 183 is sometimes albeit very briefly within 3.5 Å of the carbonyl group of benzamide.

## 3 Discussion

In order to test the correctness of the concentrations chosen for the ITC experiments, we used the (Equation 1) from Rühmann et al.

(1) $${\left[fragment\right]}_{\mathrm{cell}}=\frac{D_{\mathrm{sat}}[Protein]-[Protein]-K_{d\mathrm{fragment}}}{1-\frac{1}{D_{\mathrm{sat}}}}$$

Since the K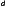 of benzoic acid is not known, we have to probe different scenarios for different ranges of affinities. Assuming a K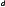 of 1 mM corresponds to a saturation ($D_{\mathrm{sat}}$) of about 80%, while a lower affinity of 10 mM saturates only 30% of the protein. As a rule of thumb, to achieve sufficient saturation of PKA or any other protein to be tested with the fragment during titration, the final concentration of the fragment added to the sample cuvette must be greater than its estimated K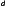 value.^[^ ^14^ ^]^ In the case of benzoic acid, the concentration used would be correct for a hypothetical dissociation constant of 1 mM, whereas for a K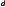 of 10 mM the fragment concentration should be higher. However, at even higher fragment concentrations, more nonspecific binding may occur. This could be an explanation why no binding is observed in any of our ITC experiments. However, we cannot exclude that due to the experimental setup, no binding is observed in the ITC. A different displacement ligand might in fact allow for detecting a binding event in the ITC. This will be covered in future studies.

In a study with the trypsin–aniline complex a protonation of the amino group of aniline takes place upon binding, demonstrated with an X‐ray/neutron diffraction structure. There, the low K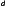 of 40 mM could be determined by ITC, due to the high solubility of aniline: high concentrations of 57 and 200 mM for displacement and low *c* value titration were applied, respectively. These concentrations exceed the aqueous solubility of benzoic acid (3.4 g L^−1^; 28 mM at 25 °C) by far.^[^ ^15^ ^]^

The different protein sources (*C.griseus* or *Homo sapiens*) should not cause a significant impact on our results, since the ITC results for the human PKA are very comparable to already published PKA arising from *C. griseus*.^[^ ^10^ ^]^ The ATP pocket is conserved (primary sequence and protein structure as well) in both species and should not impact the crystallization of the benzoic acid fragment: the six different amino acids are more than 6 Å away from the ATP pocket.

The buffer medium used for protein crystallization contains a considerable amount (see Table 1) of apolar solvents such as dimethylsulfoxide (DMSO), methanol (MeOH) and 2‐Methyl‐2,4‐pentanediol (MPD). In this environment, it is likely that the benzoic acid appears in the protonated (uncharged) form being in equilibrium with the deprotonated (charged) form. Based on our pKa measurement of benzoic acid in the X‐ray buffer, it would lead to a protonation degree of slightly below 10%. We hypothesize that already such a small amount of this protonated form of benzoic acid is sufficient to successfully soak together with PKA. However, we do not have an experimental setup to verify or falsify this hypothesis.

The pKa calculations in implicit solvent revealed a significant increase of the pKa shift of benzoic upon complexation to PKA. This was strengthened through the outcome of the MD simulations. The main result of the simulations is that the protonated and deprotonated states of benzoic acid have opposed orientations (Figure 4). The protonated state is oriented with the carboxylic acid group facing towards the protein where it is bound with hydrogen bonds to the Glu121 and Val123 amino acids (Figure 4A). These‐bonds have been highlighted by Oebbeke et al.^[^ ^9^ ^]^ and lock the ligand in its orientation and position in the binding pocket, which are maintained throughout the simulation (Figure 4B).

Glu 121 and Val 123 was also found to be important in a previous MD investigation of PKA‐ligand interactions.^[^ ^5^ ^]^ In future MD simulations it will also be instructive to directly investigate the roles of Glu 121 and Val 123 by in silico mutagenesis.^[^ ^12^ ^]^ For benzamide we find a similar binding pattern, albeit the hydrogen bond between Glu 121 and its functional group is transiently broken during the simulation unlike in the simulation of the protonated benzoic acid fragment. Deprotonated benzoic acid however is reoriented with the carboxylic acid group oriented toward the opening, where it forms hydrogen bonds with Lys72 and the Thr183 (Figure 4C). In this orientation, the functional group of deprotonated benzoic can interact with water molecular just outside the binding site. The simulations give only qualitative insight into molecular details of how PKA interacts with drug fragments. For investigating the binding free energy and quantify differences between the ligands one would need to conduct, for example, alchemical binding free energy calculations.

## 4 Conclusions

Due to the sufficiently high solubility, we were able to run two different ITC approaches (low *c* value and displacement) for the complexation of benzoic acid fragment to its target PKA. These measurements did not detect any recordable interactions.

The comparison of the buffer environment of the ITC experiment versus the protein crystallization buffer shows substantial differences. We hypothesize that the protonated form of benzoic acid is present in the crystal structure due to the chosen buffer system. The PB calculations and MD simulations strengthen this hypothesis. In contrast, the deprotonated form of benzoic acid is present when performing the ITC measurements. We conclude that the binding event cannot be measured in the ITC due to the “wrong” protonation state of benzoic acid. Furthermore, the presumably very low affinity of benzoic acid makes the appearance of a binding signal in the ITC measurement almost unlikely.

The implicit solvent pKa calculations were able to underline the protonation state of the benzoic acid complexed to PKA. The benzoic acid shows a significantly increased pKa value when complexing to its target protein PKA. The MD simulations further supports the conclusion that the protonated form of benzoic acid can bind to PKA as the X‐ray based binding pose of the protonated fragment was maintained the simulations. The deprotonated fragment preferred an alternative orientation. The MD simulations reveal similar key interactions of the protonated benzoic acid compared to the reference ligand benzamide. One can therefore assume that the (in aqueous environment rather unlikely appearing) protonated form of the benzoic acid binds to PKA.

Therefore, other approaches to rule out the effects of different buffer media are needed. Protonation effects have been demonstrated in numerous studies using ITC experiments, but this might only be suitable for ligands with an affinity $K_{d}$ <1 mM and/or a high enthalpic contribution.^[^ ^6^ ^,^ ^16^ ^,^ ^17^ ^]^ However, for low affinity fragments, high‐resolution crystallography combined with neutron diffraction structure could be used.^[^ ^18^ ^]^ In addition, orthogonal methods for the binding detection could be used as well. We envision such experiments for our future studies on PKA‐fragment complexes.

## 5 Experimental Section

5.1

5.1.1

##### Protein Preparation

For PKA, the expression and purification was performed as follows: the PKA construct was ordered from Addgene, PCR‐amplified with an N‐terminal ${\text{His}}_{9}$‐tag and an HRV 3C‐cleavage site and cloned into the pET15b plasmid with NcoI/BamHI restriction site. The construct was then transformed into chemically competent *Escherichia* *coli* Rosetta2 (DE3) and expressed. Purification of the target protein was then performed by Ni affinity chromatography, dialysis with PreScission protease, reverse Ni affinity chromatography, cation exchange and finally size exclusion chromatography. Afterward, PKA was concentrated to 15 mg mL^−1^, frozen in liquid nitrogen and stored at –80 °C until it was used again. Prior to ITC measurements, the required amount of PKA was thawed and purified again via a size exclusion chromatography column and concentrated to 10 mg mL^−1^ in 30 mM HEPES, 100 mM NaCl, 10 mM ${\text{MgCl}}_{2}$ at pH 7.4.

##### ITC

The *c* value is defined as $c=\frac{{\left[M\right]}_{0}}{K_{d}}$ with ${\left[M\right]}_{0}$ being the concentration of the titrand and should ideally be between 1 and 1000 for reliable determination of the dissociation constant.^[^ ^19^ ^]^ This means that there is both an upper limit and a lower limit for the determination of the thermodynamic parameters. Low *c* value titration was feasible and gave valid *K* 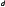 values, but it required a lot of both fragment and protein and errors regarding ΔH were likely.^[^ ^14^ ^]^

Displacement titrations have been carried out for weak binding fragments as well as for ligands with a high affinity in the one‐digit nanomolar range. The displacement experiment for compounds showing low affinity toward the target required the presence of a high affinity ligand that binds to the same binding site as the weak one. Prior to the titration, the protein was incubated with the fragment and in the following ITC experiment, the strong ligand displaced the weak binder from the binding site. The displacement titration as well as the one with the strong ligand yield affinities ($K_{\mathrm{observed}}$ for the displacement titration and $K_{\mathrm{reference}}$ for the titration with just the ligand) that were used to calculate the $K_{\mathrm{fragment}}$ of the weakly binding molecule (Equation 2).

(2) $$K_{\mathrm{fragment}}=\left(\frac{K_{\mathrm{reference}}}{K_{\mathrm{observed}}}-1\right)*\frac{1}{{\left[\mathrm{fragment}\right]}_{\mathrm{cell}}}$$

ITC measurements with PKA were carried out in a buffer containing 30 mM HEPES, 100 mM NaCl, 10 mM ${\text{MgCl}}_{2}$, pH 7.4, 2% (v/v) DMSO) at 25 °C. The titration protocol consisted of 19 injections, the first of which was 0.4 μL, followed by 18 titrations of 2 μL each.

All measurements were conducted on a MicroCal PEAQ‐ITC Automated ITC (Malvern Panalytical). The obtained thermogram peaks of all titrations were integrated and fitted with MicroCal PEAQ‐ITC Analysis Software 1.41. The dissociation constant K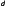 and the enthalpy ΔH of the fragments was determined as previously described.^[^ ^20^ ^]^

##### pKa Measurements

The UV–vis and pH measurements for determining the pKa values were performed using a Sirius T3 Pion instrument. The titrations were performed as triplicates with the titration method low to high at T = 25 °C. Both, the soaking buffer and the ITC buffer without the addition of benzoic acid were measured as references, respectively. The reference and sample V = 30 μL. The obtained pKa value corresponds to the mean value of the single titrations.

##### Implicit Solvent pKa Calculations

For protein pKa calculations, a program from OpenEye based on the Zap finite difference PB solver was used.^[^ ^21^ ^,^ ^22^ ^]^ For the partial charges of the protein, Delphi radii and CHARMM36 all‐hydrogen partial charges were used, and the am1bccsym method was used to assign appropriate charges to the benzoic acid.^[^ ^23^ ^]^ The benzoic acid fragment outside the hinge was removed from the structure. The pKa of the ligand's carboxylic group was set to 4.2 according to the experimental value.^[^ ^24^ ^]^ An inner dielectric of 15, an ionic strength of 0.05 M, and an ionization (i.e., pH) of 6.9 were used.

##### MD Simulations

Ligands were parameterized for CHARMM36^[^ ^25^ ^]^ using CGgenFF.^[^ ^26^ ^]^ The CHARMM TIP3P water model and the CHARMM36 protein force field were used.^[^ ^27^ ^,^ ^28^ ^]^ Production simulations were ran in GROMACS^[^ ^29^ ^,^ ^30^ ^]^ for apo‐PKA, PKA with benzoic acid (PDB entry: 6SNN), PKA with deprotonated benzoic acid and benzamide (PDB entry: 6SNX) for 1 μs each after two 100 ps equilibration simulations in the NVT and NPT ensemble, respectively.

The simulations were performed in steps of 2 fs, using the leap frog integrator. The temperature was kept at 300 K with the Bussi–Donadio–Parrinello velocity rescaling thermostat with a time constant of 2.0 ps.^[^ ^31^ ^]^ The pressure was kept at 1 bar with an fully isotropic Parinello–Rahman barostat that used a time constant of 5 ps and a compressibility of 4.6E–5 1/bar.^[^ ^32^ ^,^ ^33^ ^]^ The whole simulation setup included the PKA protein, the ligand, 22.204 water molecules and one chloride ion to neutralize charge. The total amount of simulated atoms was 72230. An additional simulation with a NaCl concentration of 150 mM was run for PKA in complex with deprotonated benzoic acid for a duration of 2 μs.

Trajectories were analyzed with MDAnalysis^[^ ^34^, ^35^ ^]^ and with VMD.^[^ ^36^ ^]^

## Conflicts of Interest

The authors declare no conflict of interest.

## Supporting information

Supplementary Material

## Data Availability

The simulation trajectories are uploaded on zenodo: DOI: 10.5281/zenodo.15100325.
