# Supplementary material for: Proton First: Rationalizing a Proton Transfer in a Protein‐Fragment Complex
Source: ChemMedChem. 2025 Sep 14;20(20):e202500244. doi: 10.1002/cmdc.202500244 (PMC12530840; doi:10.1002/cmdc.202500244)
Supplement: Supplementary file 1 — Supplementary Material [file CMDC-20-e202500244-s001.pdf]

Supporting Information

Flexibility of the Proteins in Molecular Dynamics Simulations

In our atomistic molecular dynamics simulations, we track how a ligand is changing the 3D structure and conformational flexibility of a protein. In order to gather this information, we calculated the displacement averaged over all residues from the initial structure as the root mean square distances (RMSDs). The conformational flexibility is measured by the root mean square fluctuations (RMSFs), which calculates the time averaged fluctuation of every single residue from the average structure. For both measures the trajectory is aligned to a reference structure, here taken to be the initial configuration.

In absence of a ligand at the binding site, there are some local structural rearrangements in PKA as shown by the RMSD to the initial structure (see Figure S1). The increasing RMSD indicates local structural rearrangement. For both deprotonated and protonated benzoic acid at the binding site, little rearrangement of the protein backbone from the initial state is seen (Figure S1 B,C). The ligand RMSD on the other hand differs strongly between the protonation states. The protonated benzoic acid fluctuates stable about 2 ˚A around the crystal structure, while the deprotonated state has more then 4 ˚A deviation. This RMSD results fit well to the reorientation of the deprotonated benzoic acid as ligand in PKA (Figure 4).

The analysis of the RMSF shows that the most flexible region of the protein without any ligands is in the segment of residues 316 to 331 (see Figure S2, dark blue area) with a peak RMSF of 8 ˚A. Simulations with benzoic acid in both protonation states show overall the lowest RMSF values throughout the whole protein compared to the other simulation setups including simulations without a ligand. The RMSF comparisons with the ligand free protein show that the activation loop is partially stabilized by protonated and deprotonated benzoic acid while it gets destabilized by benzamide (Fig S2). The residues 316 to 334, which we refer to as the outer hinge loop, is getting stabilized by all ligands. It further shows that the activation loop (resid 191 to 205) gets destabilized by benzamide.

A B

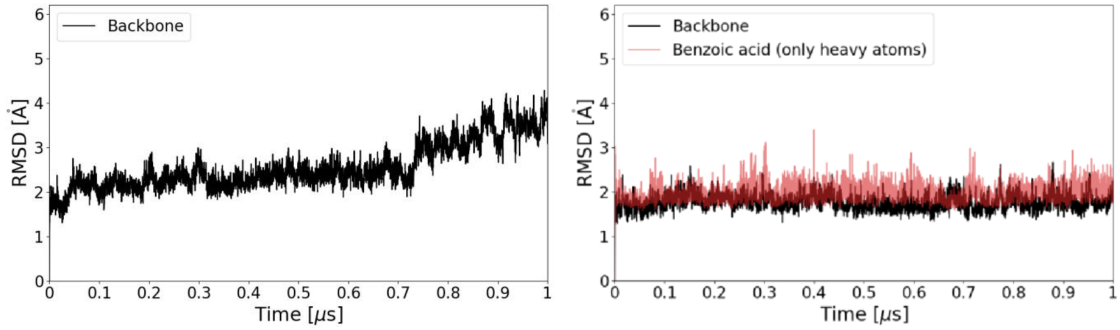

C

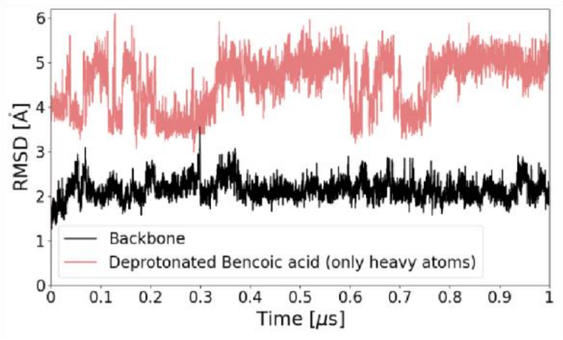

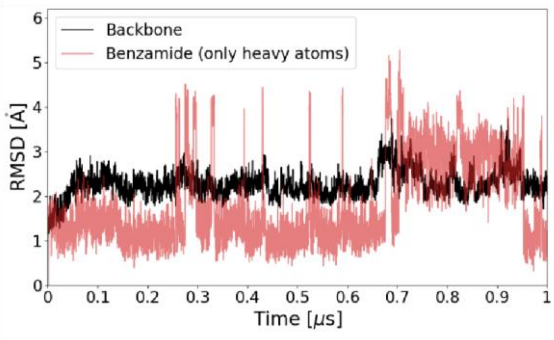

D

Figure S1: Conformational similarity to reference structures as judged by RMSD.A) Backbone RMSD to X-ray structure 6SNN for simulation with no ligand in the ATP binding site. B) Simulation with protonated benzoic acid fragment. Backbone RMSD (for PKA) and heavy atom RMSD (for fragment) to PDB entry 6SNN are shown. C) Simulation with de-protonated benzoic acid fragment. Backbone RMSD (for PKA) and heavy atom RMSD (for fragment) to PDB entry 6SNN are shown. D) Simulation with benzamide fragment. Backbone RMSD (for PKA) and heavy atom RMSD (for fragment) to PDB entry 6SNX are shown.

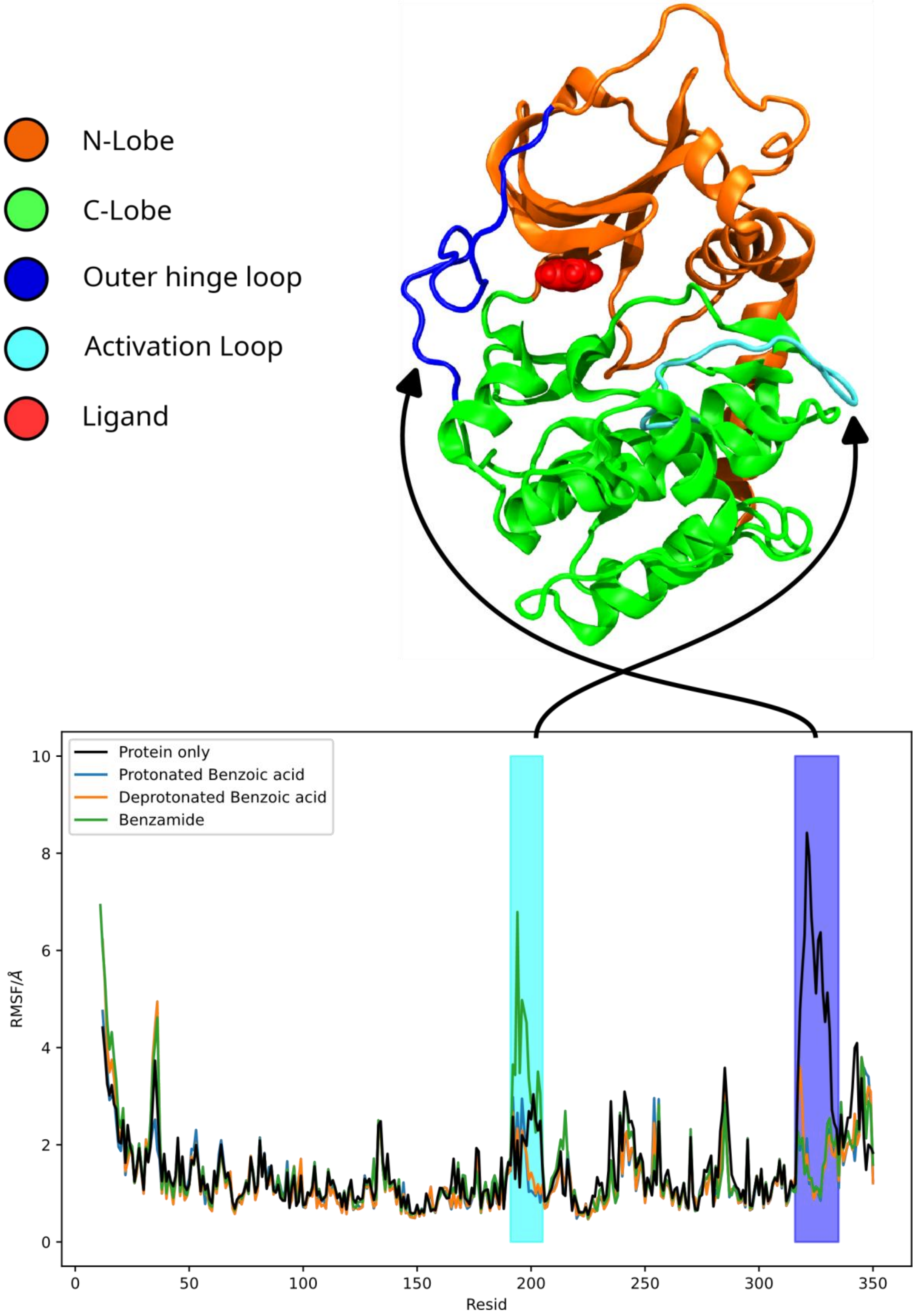

Figure S2: Root Mean Square Fluctuation of the Protein PKA for the Ligands protonated benzoic acid, deprotonated benzoic acid, benzamide as well as no ligand. The areas with mayor change, namely the activation loop (resid 191 to 205) and the outer hinge (resid

316 to 335) are marked in the RMSF plot and reference to the corresponding part of the protein.

9 Additional simulation of deprotonated benzoic acid in complex with PKA

A simulation with a salt concentration of 150 mmol was performed. Fig S3 shows how the mininmum distance between Lys 72 and the ligand changes over time and that the ligand is bound for about 0.5 μs until it dissociates from PKA. The ligand binds to a different binding pocket outside the active site for 0.93 μs. Tracking the distance between Lys 72 and visualization further supports the assumption that deprotonated benzoic acid is only a weak Binder to PKA.

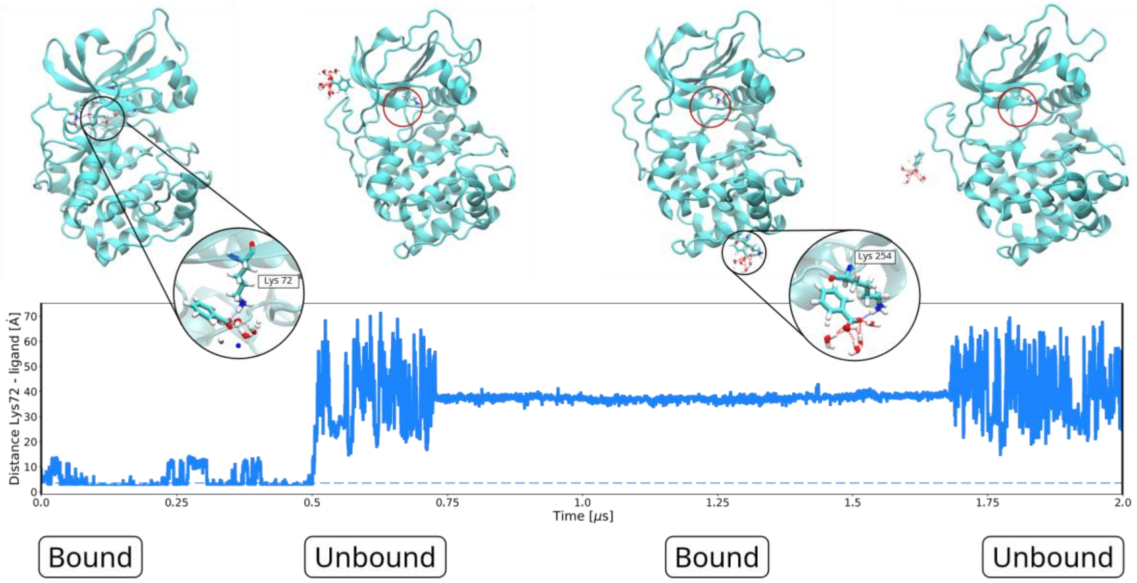

Figure S3: Interactions of deprotonated benzoic acid with PKA in additional molecular dynamics simulation trajectory. PKA in complex the deprotonated benzoic acid was investigated in a simulation with a salt concentration of 150 mmol. The protein snapshots indicate the ligand position at the different areas of the simulation. The minimum distance between Lys 72 and the ligand is shown to track the interactions of deprotonated benzoic acid and PKA.

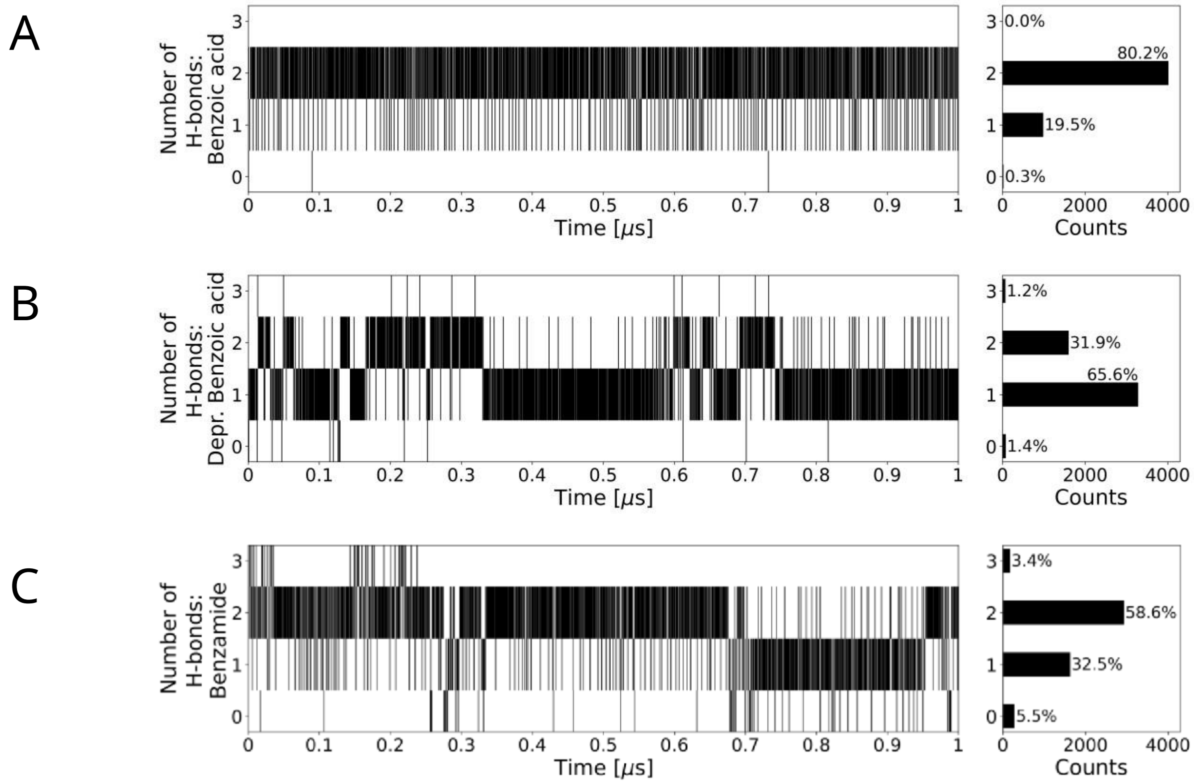

Figure S4: Number of hydrogen bonds over time in the simulations of PKA with fragments.

10 MMPBSA

Since the hbond analysis led only to a shallow understanding of the varying binding behaviours, an additional molecular mechanic poisson boltzmann surface area (MMPBSA) analysis was performed. MMPBSA predicts a binding energy by separating the binding energy in several terms like molecular mechanics energy, PoissonBoltzmann energy and Van der Waals energy and more. Based on this, the binding energy and the distributions from different ligands can be approximated[13].

In the case of protonated benzoic acid the strongest contributed is from the GLU121 and additional binding contributions are from VAL57, MET120, GLU121, TYR122, VAL123, LEU173 and PHE327, while LYS72 and THR183 have a slightly antibinding contribution. The average binding enthalpy results in −16.1 ± 0.1 kcal/mol, fluctuating with a standard deviation of 2.2 kcal/mol. This is in strong contrast to deprotonated benzoic acid, where the strong binding residue is LYS72 (which was antibinding before) supported by VAL57, VAL104, MET120, LEU173 and ASP183. The antibinding contributions from the deprotonated state result from GLU121 (which was the strongest binding contribution before), GLU127, GLU170 and ASP184 which results in an overall positive predicted binding free energy. The deprotonated state results in an average binding enthalpy of −6.5 ± 0.3 kcal/mol, fluctuating with a standard deviation of 5.3 kcal/mol. A more precise method to calculate the binding free energy would be to apply alchemical relative binding free energy which turned out to not be trivial. To fully understand these mechanisms, more research has to be done.

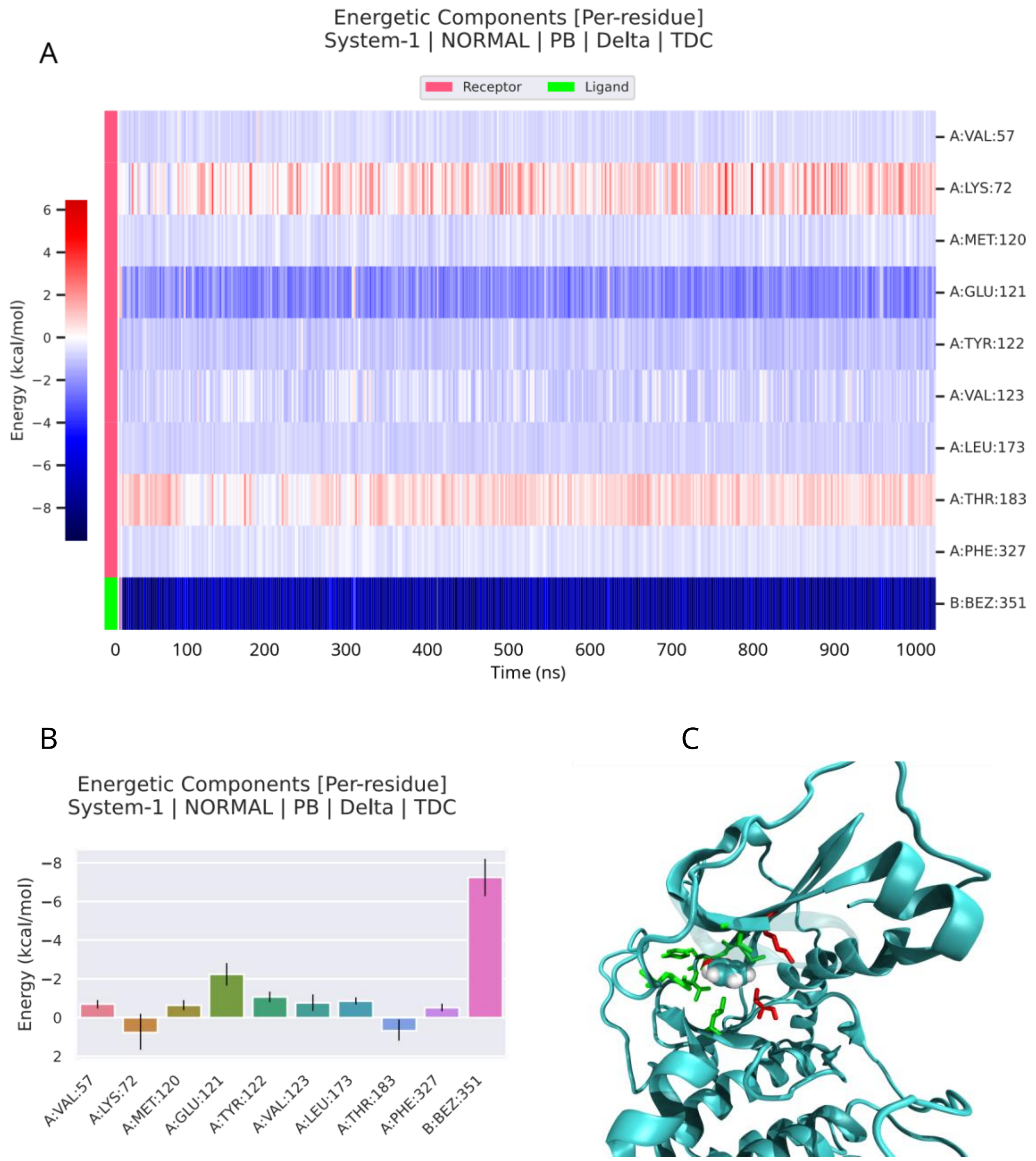

Figure S5: MMPBSA analysis of protonated benzoic acid. Contributions of the different ligands to the binding free energy over time(A). Resulting Energy values in timeaverage(B) and the ligand position and orientation(C). The ligands in latter are colored green for the binding contributions and red for the unbinding distributions.

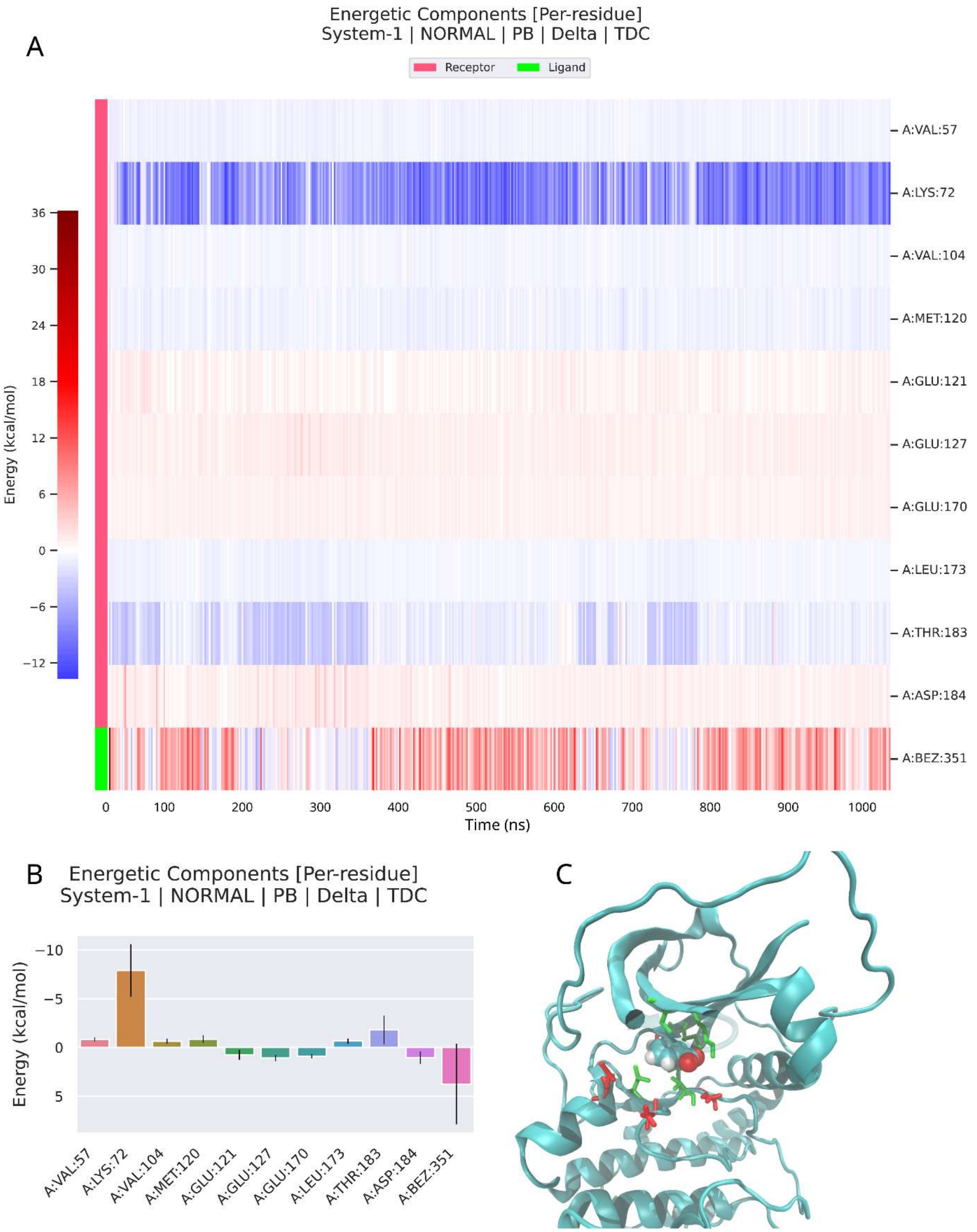

Figure S6: MMPBSA analysis of deprotonated benzoic acid. Contributions of the different ligands to the binding free energy over time(A). Resulting Energy values in timeaverage(B) and the ligand position and orientation(C). The ligands in latter are colored green for the binding contributions and red for the unbinding distributions.
